# Gender differences in health care use among the elderly population in areas of Norway and Finland. A cross-sectional analysis based on the HUNT study and the FINRISK Senior Survey

**DOI:** 10.1186/1472-6963-6-110

**Published:** 2006-09-04

**Authors:** Anna Liisa Suominen-Taipale, Tuija Martelin, Seppo Koskinen, Jostein Holmen, Roar Johnsen

**Affiliations:** 1 Department of Public Health and General Practice, Norwegian University of Science and Technology, Trondheim, Norway, and Department of Health and Functional Capacity, KTL (National Public Health Institute), Mannerheimintie 166, FIN-00300, Helsinki P.O. Box 5281, Finland; 2 Department of Health and Functional Capacity, KTL (National Public Health Institute), Helsinki, Finland; 3 Department of Health and Functional Capacity, KTL (National Public Health Institute), Helsinki, Finland; 4 HUNT Research Centre, Department of Public Health and General Practice, Norwegian University of Science and Technology, Verdal, Norway; 5 Department of Public Health and General Practice, Norwegian University of Science and Technology, Trondheim, Norway

## Abstract

**Background:**

The aim of the study was to examine gender differences in the self-reported use of health care services by the elderly in rural and metropolitan areas of two Nordic countries with slightly different health care systems: Finland and Norway.

**Methods:**

Population based, cross-sectional surveys conducted in Nord-Tröndelag Norway (1995–97) and in rural and metropolitan areas of Finland (1997) were employed. In the Norwegian data, a total of 7,919 individuals, aged 65–74 years old were included, and the Finnish data included 1,500 individuals. The outcome variables comprised whether participants had visited a general practitioner or a specialist, or had received hospital care or physiotherapy during the past 12 months. Gender differences in the use of health care services were analysed by multiple logistic regression, controlling for health status and socio-demographic characteristics.

**Results:**

In Norway, elderly women visited a specialist or were hospitalised less often than men. In Finland, elderly women used all health care services except hospital care more often than men. In Norway, less frequent use of specialist care by women was not associated with self-reported health or chronic diseases.

**Conclusion:**

The findings revealed differences in self-reported use of secondary care among different genders in areas of Norway and Finland.

## Background

In the Andersen-Nyman model [1], the use of health care services is defined as a function that includes three types of explanatory factors: measures of need including self-rated health, and non-health factors that either predispose or enable the individual to obtain care. In this model, the patient's gender is identified as one of the predisposing factors, and gender difference in service use is assumed to indicate the differing predisposition of men and women to seek care. The historical focus of studies into service use has been on need. Gender is often included in multivariate analyses on health care use, but it often serves as a potential confounder rather than an explanatory variable [2]. It is well documented that being female predisposes to obtaining primary health services. Research analysing gender differences in the use of other types of health services is scarce, and this is especially the case in elderly populations. Additional information is required as previous evidence exists of a gender bias, for example, in the management of coronary heart disease [3], accessibility to short-stay and emergency hospital services [4,5], and waiting times in emergency rooms in favour of men [6]. Older people are paradoxically often excluded from studies on health care use, since the need for health care services increases with age. Studies that include the elderly are therefore necessary to identify factors which determine the use of health care. Information on the under use of health care in elderly is important, as good care is likely to improve the quality of life, increase the number of years without functional limitations and diminish existing health differences.

The aim of this study is to examine gender differences in the use of several types of health services among elderly populations in distinct, rural and metropolitan, areas of Norway and Finland. The study employs data from the Nord-Trondelag Health Study (1995–97) and the FINRISK Senior Survey (1997). Norway and Finland are two Nordic countries that have similar population characteristics, culture and a similar social structure. Yet minor, but distinct, differences in the supply and organisation of health care exist at different levels. These include the gender distribution of the medical profession and the fact that physicians in Finland often work simultaneously in the private and public sector, whereas in Norway physicians work in one or the other. Therefore, it is of particular interest to compare gender differences in the use of different levels of health care in Norway and Finland.

## Methods

The data were obtained from two cross-sectional surveys: one conducted in Norway in 1995–97 (*The Nord-Trondelag Health Study, HUNT*), and the other in Finland in 1997 (*FINRISK – senior survey*).

The HUNT survey was focused to all residents aged 20 years or over in the Nord-Tröndelag county and included health examination and questionnaires. Nord-Tröndelag is located in the central part of Norway, and the geographical, demographic and occupational structure is fairly representative of Norway as a whole. The Norwegian data included in this study were gathered through two postal questionnaires. The first one included questions of socioeconomic factors, self-reported health and morbidity, and was sent prior to attending a health screening. The other one included questions of use of health care service and was delivered after the examination, to be filled in at home and returned by mail. Of the age group 65 – 74 years (n = 11092) only those individuals who returned both questionnaires were included (n = 8599). Those who had incomplete answers on the questions on use of health care services (n = 672) or reported living permanently in a hospital (n = 8) were excluded. The final data comprised 7,919 individuals representing 71% of all the 65–74 year-old residents in the county.

The FINRISK senior survey was composed from a medical examination, questionnaires and an interview. Women and men aged 65–74 were included in a random sample in two administrative areas in Finland; the Helsinki Metropolitan Area in the south of the country and the rural region North Karelia in the north-east. The combination of these two areas can be regarded to represent the whole country. The sample size was 1500, consisting of 250 women and 500 men in both areas in 1997. Individuals reporting to live in institutions (n = 5) were excluded. The response rate was 86% (n = 1,283). The data presented here were gathered by a self administered questionnaire and an interview. The postal questionnaire including questions of socioeconomic factors was sent together with an invitation to a medical examination. Questions concerning self-reported health status and morbidity, and use of health care services were posed during a personal interview at the examination site. Detailed descriptions of the surveys are presented elsewhere [7,8].

### Use of health care services

The use of health care services in the Norwegian data was elicited by asking the questions 'Have you, during the previous 12 months, visited a general practitioner, occupational doctor, doctor in a hospital, some other doctor, or a physiotherapist?' and 'Have you been hospitalised during the last 5 years?' In the Finnish data, the questions were 'Have you used the following health care services during the previous 12 months?' The alternatives listed were hospital care, specialist care, general practitioner care and physiotherapy/rehabilitation.

In Norway, visits to a general practitioner (75% of the respondents) were combined with visits to an occupational health service (11% of the respondents) or some other doctor (13% of the respondents) because these were all considered to represent visits to a primary care physician. In the Norwegian data, visits to a doctor in a hospital during the last 12 months included visits to hospital outpatient clinics. Furthermore, visits to a physician included visits to a general practitioner, a specialist or both during the previous 12 months in both data sets.

Five dichotomous response variables were formed for the analyses: having visited 1) any physician, 2) a general practitioner (GP), 3) a specialist, 4) a physiotherapist during the past 12 months; and 5) a hospital during the past 5 years in Norway and during the past 12 months in Finland (yes = 1, no = 0).

### Explanatory variables

Table 1 presents the distribution of the explanatory variables according to gender with the patient's gender being the main point of interest. Two indicators of need were included; self-rated health and self-reported chronic diseases. The survey in Norway posed a single question about the respondents' self-rated health status: 'How do you consider your health at the moment?' with response options of 'poor, fair, good or very good'. In Finland the question was 'How good would you say your health is at the moment?' with response options of 'Poor, fairly poor, fair, fairly good, good'. The answers were grouped into two categories for further analyses: 'good health' (very good, good or fairly good), and 'fair or poor health' (fair, fairly poor or poor).

In the analyses, self-reported chronic diseases were combined into seven groups: 1) cardiovascular diseases, 2) musculoskeletal disorders, 3) respiratory diseases, 4) mental disorders, 5) diabetes, 6) cancer, and 7) other somatic diseases. In Norway, cardiovascular diseases included myocardial infarction, angina pectoris, cerebral stroke and high blood pressure; musculoskeletal disorders included osteoporosis, fibromyalgia, rheumatoid arthritis, arthrosis, Bechterews syndrome and other musculoskeletal diseases; respiratory diseases included bronchial asthma; other diseases included high/low metabolism, goiter, other diseases of the thyroid gland, epilepsy and other illness. In Finland, the cardiovascular group included myocardial infarction, angina pectoris, cerebral stroke, high blood pressure, thrombosis in lower extremities and heart insufficiency; musculoskeletal diseases included rheumatoid arthritis, arthrosis, some other disease of the joints or back disease; respiratory diseases included bronchial asthma and emphysema; other diseases included peptic ulcer, gall stones and urinary infection.

In the study we included age group, level of education and marital status as individual factors predisposing to and/or enabling the use of health care. In addition, a dichotomous variable indicating the region of residence was included in Finland (North Karelia, Helsinki Metropolitan Area). The level of education, instead of occupational class, was chosen to represent an individual's socioeconomic position, since nearly all the respondents were pensioners. The level of education was classified as primary school, middle level education and university degree in both data sets. A four class classification for marital status was used: single, married or cohabiting, divorced or separated, and widowed.

### Statistical methods

The distributions of the explanatory variables were first described by country and gender, and gender differences were tested by means of a chi-square test (Table 1). The reported visiting frequencies in various health care services were described by country, sex, and five-year age group, and sex differences were tested with a chi-square test.

As a first step in the explanatory analyses, bivariate analyses based on contingency tables and chi-square tests were first performed to analyse the association between each explanatory factor and visit to a health care service. Because each variable significantly contributed to the explained variance in one or both data sets, they were all chosen to be included in the multivariate logistic regression models. A logistic regression model was used to assess adjusted differences in the use rates between genders (Table 2). Gender, as the main explanatory variable, together with age group and region (only in Finland) were first added to the model. Of the potential confounders, self-rated health and self-reported chronic diseases were then added to the model, followed by the causally more remote factors, such as level of education and marital status. Interactions between gender and all the explanatory variables were tested one by one in the model including all the explanatory variables (Model 4 in Table 2). In Norway, significant interactions (p < 0.01) between sex and age group were detected for visiting a physician and a general practitioner, and in Finland for visiting a physiotherapist. Analyses stratified by age group were therefore also performed (Table 2). In the Norwegian data, minor interactions (0.01< p <0.05) between gender and some other variables were found, too.

## Results

As shown in Table 1, Norwegian men more frequently rated their health as good compared with women. In Finland, there were no differences between the genders concerning self-rated health status. On the whole, the Finnish elderly population reported having poor health and cardiovascular or musculoskeletal diseases more often than their Norwegian counterparts. Otherwise, the panorama of chronic diseases was similar in Norway and Finland. Gender differences in the prevalence of self-reported diseases were quite similar in both countries, with women reporting musculoskeletal, mental and other diseases more often, while men reported cardiovascular diseases more often.

**Figure 1:**
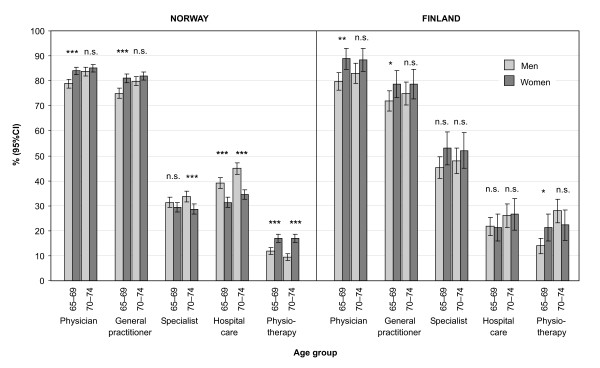
**Reported visiting frequencies in elderly in areas of Norway and Finland; Reported visiting frequencies in 65–74-year-old women and men in Norway in 1995–97 and in Finland in 1997**. (*p < 0.05, **p < 0.01, ***p < 0.001 refer to chi square tests between the genders). ^1 ^Nord-Tröndelag in Norway, Helsinki Metropolitan area and North-Karelia in Finland

A greater proportion of women than men consulted any type of physician or general practitioner both in Norway and in Finland; in Norway, this difference was statistically significant only in the age group 65–69 (Fig 1). Norwegian women (in the age group 65–69) visited a GP and a physiotherapist more often than men but visited a specialist or received hospital care less often. In Finland, women visited any physician, general practitioner or specialist more frequently. A similar difference was also found in visits to a physiotherapist in the age group 65–69, while Finnish men aged 70–74 used physiotherapy services slightly more often than women. No gender difference in hospital care was found in Finland.

All explanatory variables were associated with the use of health care. The use of health services was particularly common in persons who reported poor self-rated health or any chronic diseases. However, gender differences in the use pattern remained even after adjusting for health status and socio-demographic variables (Table 2). Regardless of these factors, Norwegian women were less often referred to secondary care compared with men.

A few interactions between gender and the other explanatory variables were detected in Norway. The gender difference in the use of specialist or hospital care in Norway was slightly more accentuated among those with cardiovascular disease than those not reporting such a disease. In addition, elderly Norwegian women with cancer had visited any physician or a general practitioner less often than male cancer patients, while the opposite was true of those not reporting cancer. On the other hand, the more frequent use of physiotherapy services among Norwegian women compared with men was accentuated among cancer patients. However, among elderly widowed respondents there was no gender difference in the use of physiotherapy services, and the gender difference in the use of hospital care was larger among those with a university degree than among those with primary level education only.

## Discussion

Our analyses revealed that in Norway, elderly women visited a specialist or were hospitalised less often compared with men. In Finland, elderly women visited any physician, general practitioner or specialist slightly more often than men; in addition, Finnish women aged 65–69 visited a physiotherapist more often than men. Gender differences were not associated with self-reported health status, the type of chronic condition, or socio-demographic characteristics.

The present study employed two carefully designed large population based surveys with high response rates. The comparability between the two surveys was presented earlier and was assessed as being quite good [9]. Data collection followed a basically similar pattern and the main outcome variables were comparably measured. However, information on the use of specialist care may not be exactly congruent as in Finland specialist care included visits to specialists working in hospitals and visits to physicians working as private practitioners whereas in Norway, only visits to physicians working in hospitals were included. Also information concerning use of hospital care is not quite consistent due to different time periods. There were some differences between the Norwegian and Finnish survey methods as the questions concerning health service use in the Norwegian data was presented in the second questionnaire after health examination, while the questions to participants in the FINRISK were posed during and interview just prior to the medical examination. Different data collection methods largely explains the little lower response rate in Norway which could cause non response bias, especially in elderly populations. However, some reports [10] have indicated that low response rate does not necessarily result in non-response bias and no general rule regarding the effects of non-response can be formulated [11]. Besides, response rate also in the Norwegian data is regarded as good. Picavet [12] compared mail and interview methods in national surveys and concluded that for many health topics, such as use of health care services, carefully designed mailed surveys are probably an equally good alternative for interview studies. In his study respondents to mail surveys report higher figures on use of health care but this was not shown in our study as there were hardly any difference between the overall visiting rates to a physician. In addition to overall estimates of self-reported health, this study simultaneously enables self-reported morbidity to be accounted for even though the disease groups were not fully identical in the surveys. As a whole, self-report is shown to be reasonably accurate method for obtaining health care utilization data also in community-dwelling seniors [13].

### Higher use of health care services by women

In Norway, elderly women sought care from a GP or a physiotherapist more frequently than men, particularly in the 65–69 age group. In Finland, visiting rates among women were higher for all care (except hospital care and, for the over 70, a physiotherapist) even after adjusting for self-reported health and chronic diseases. In addition to more frequent medical needs, several explanations for the higher use of health care services among women have been suggested. Women are assumed to more easily adopt the sick role; they tend to recognise and experience more health problems than men, because it is socially and culturally acceptable for women to be ill and seek professional help [14]. The higher rates of consulting among women reflect their greater awareness and concern about health-related problems [15], underlying gender-related psycho-social and behavioural influences [16], or perception of symptoms [17-20].

In addition, a gender difference in the general willingness to consult has sometimes been suggested as an important cause for differences in the use of health care services. However, some studies do not support this explanation. Accordingly, there is a great commonality in how men and women react to common bothersome symptoms [21], and women do not have a greater propensity to consult than men once the symptoms are perceived [22,23]. Hunt et al. [24] also argue against the idea that women are simply more likely to consult a GP irrespective of the underlying morbidity. In adults over 65, higher rates are due to the perceived severity of the condition [25]. However, as noticed by Hunt et al. [24] reports of pain, limitation, the experience of a condition and the meaning attached to these are not unproblematic, and are probably the product of complex social and cultural processes which may or may not be patterned according to gender. Differences in mortality between the genders could also be one explanation. Men's life expectancy is shorter than women's and the healthiest men survive. They may simply not need as many health care services as elderly women. The more prevalent use of physiotherapy services among Finnish men aged 70 or over compared with women was also observed in the Health 2000 Health Examination Survey [26]. This pattern is likely to be due to the fact that men in these cohorts participated in World War II and they are therefore entitled to veterans' rehabilitation services.

### Gender differences in the use of secondary care

It has also been reported earlier that adult, often older, men are referred for specialty care [27] or hospitalised [28,29] more often than women. Mutran and Ferraro [29] have suggested that the reason for this is the nature of elderly men's illnesses (e.g. cardiovascular and respiratory diseases), in contrast to the problems experienced more often by women, i.e. men suffer from more severe and complicated health problems. The fact that Norwegian women declared poorer health, sought primary care more often but received specialist care less often would support an argument that women are healthier but have worse perceptions of their own health. It has been suggested that women suffer from non-fatal conditions that can be taken care of by primary care more often than men. However, as the gender difference remained even after controlling for self-rated health or chronic conditions, the explanation cannot be that simple. No matter what disease Norwegian elderly women reported, they were referred to specialist care less often than men. In fact, the findings suggest that the gender difference was even accentuated among those reporting a cardiovascular disease or cancer.

### Role of the patient's and physician's gender

GPs make most referrals due to a combination of medical and nonmedical reasons [30-32]. Variations in the referral patterns of primary care physicians have been linked both to the patient and the physician, even though the influence of these provider-related factors has remained largely unexplored [34]. Clinical decision-making is based on the verbal report of the patient, and at its best this communication leads to a mutual understanding. In a recent study by Little et al. [35] perceived patient pressure experienced by a physician appeared to be a strong independent predictor of all doctors behaviour such as making a referral. However, Britten [36] argues that these perceptions may be overemphasized in the doctor's mind, i.e. be incorrect. Gender roles probably have an impact on the dynamics and interaction during consultations.

Scott et al. [30] reported earlier that the patient's gender may influence a GP's decision-making. In Denmark, disagreement about the content of a consultation between a patient and a GP was observed more often when the patient was female [37]. Recently Bertakis et al.[38] stated that patient variables, including gender, appear to be more important in explaining referrals than was recognized in previous research. Mutran and Ferraro [29] also suggested that among elderly persons, the physician's perception of male versus female patients could contribute to differences in hospitalisation. Physicians may think that men are not able to care for themselves at home in the way that women do, and men are less likely to be seen as hypochondriacs, or that emotional factors contribute to health problems more strongly in women than in men [39]. Hospitalisation may therefore be based not only on biological but also on social factors, as physicians perceive and treat male and female patients differently. The initial complaints and presentations by men and women may be very similar, but women may be misinterpreted more easily leading to a situation where women and men with similar problems end up with dissimilar diagnoses and treatment.

Female doctors are said to be more sensitive to women's problems than male doctors [40] and female physicians are more likely to see female patients [41,42]. In Finland, an exceptionally high proportion (58 %) of female doctors were already working as GPs in health centres [43] at the time the study took place. In Norway, GPs were more often male (72 %) [44]. This may contribute to the demonstrated differences between Norway and Finland.

The effect of patient's female gender is possibly mediated by educational level. Fylkesnes et al. [45] have earlier reported that among adult population access barriers to specialist care in Norway exist and relate to social status. In the present study educational level of Norwegian women was lower compared to Finnish women or men in both countries. As those higher educated are supposed to have more communicative skills they are thus more competent in their arguments for specialist care. Further, lack of significant gender-based differences in Finland may also due to overall easier access to specialist care as supply of private services is larger and most private practitioners are specialist. Patient can easily contact a specialist without consultations to a GP or referral. Cultural differences in health awareness and help-seeking behaviour probably play a role as well.

## Conclusion

Elderly women in Nord-Tröndelag Norway declared themselves as having a worse health status than men, and could thus be expected to have a greater need for all health care. However, in contrast to elderly women in rural and metropolitan areas of Finland, they reported lower use of specialist and hospital care in spite of need. Norwegian women's lower educational level as well as easier access to specialist services in Finland probably have effect. Restricted access to secondary care among elderly women may have financial and health consequences leading to even worse health and an impaired quality of life.

## Competing interests

The author(s) declare that they have no competing interests.

## Authors' contributions

ALS-T drafted the manuscript and performed the statistical analysis.

TM has been involved in drafting the manuscript and interpretation of data, and revised the manuscript critically.

SK has been revised the manuscript critically and made substantial contributions to acquisition of data in the FINRISK Senior Survey.

JH has been revised the manuscript critically and made substantial contributions to acquisition of data in the HUNT Study.

RJ has been revised the manuscript critically and made substantial contributions to conception and design of the present study, and acquisition of data in the HUNT Study.

All the authors have read and approved the final manuscript.

## Pre-publication history

The pre-publication history for this paper can be accessed here:



## Figures and Tables

**Table 1:** Distribution of 65–74-year-old men and women according to age, educational level, marital status, self-reported health and chronic diseases in areas ^1^of Norway (1995–97) and Finland (1997).

		NORWAY		FINLAND	
		Men	Women	Men	Women
		n = 3 732	N = 4 187	n = 855	n = 428
		n (%)	n (%)	n (%)	n (%)
Age		n.s.^2^		*	
	65 – 69 years	1 837 (49)	2 069 (49)	497 (58)	221 (52)
	70 – 74 years	1 895 (51)	2 118 (51)	358 (42)	207 (48)
Educational level		***		n.s.	
	Primary school	1 898 (57)	2 735 (76)	529 (63)	264 (63)
	Middle level ^3^	1 133 (34)	618 (17)	243 (29)	132 (32)
	University degree	327 (10)	232 (6)	73 (9)	23 (5)
Marital status		***		***	
	Married/cohabiting	2 924 (78)	2 573 (62)	663 (78)	183 (43)
	Single	373 (10)	187 (4)	77 (9)	45 (11)
	Divorced/separated	156 (4)	124 (3)	63 (7)	43 (10)
	Widow	273 (7)	1 296 (31)	52 (6)	157 (37)
Self-rated health		***		n.s.	
	Good	2 230 (60)	2 251 (54)	347 (41)	184 (43)
	Fair or poor	1 480 (40)	1 894 (46)	507 (59)	243 (57)
Self-reported chronic disease		***		*	
	Cardiovascular	1 546 (41)	1 539 (37)	453 (53)	198 (46)
		***		**	
	Musculoskeletal	919 (25)	1 737 (41)	454 (53)	266 (62)
		**		n.s.	
	Respiratory	384 (10)	357 (9)	111 (13)	53 (12)
		***		***	
	Mental	203 (7)	507 (12)	49 (6)	48 (11)
		n.s.		n.s.	
	Diabetes	268 (7)	273 (7)	92 (11)	38 (9)
		**		n.s.	
	Cancer	253 (7)	357 (9)	47 (6)	20 (5)
		***		***	
	Other	557 (15)	895 (21)	65 (8)	64 (15)
Region				n.s.	
	North Karelia			459 (54)	225 (53)
	Helsinki Metropolitan area			396 (46)	203 (47)

^**1 **^Nord-Tröndelag in Norway, Helsinki Metropolitan area and North-Karelia in Finland

^**2 **^χ^2^-test for the gender difference: n.s. = not significant, * p < 0.05, ** p < 0.01, *** p < 0.001

^**3 **^Includes junior high school, senior high school and vocational training

**Table 2:** Sex differences in having visited a health care professional or received hospital care during the previous year (odds ratios for women with 95% confidence intervals, men = 1) among 65–74-year-old persons in areas ^1 ^of Norway (1995–97) and Finland (1997).

		NORWAY OR (95% CI)				FINLAND OR (95% CI)			
		Model 1	Model 2	Model 3	Model 4	Model 1	Model 2	Model 3	Model 4
VISITS TO A PHYSICIAN									
Total		1.3 (1.1–1.4)	1.2 (1.04–1.3)	1.1 (1.01–1.3)	1.3 (1.1–1.5)	1.8 (1.3–2.6)	1.9 (1.2–2.7)	2.0 (1.3–2.9)	2.1 (1.4–3.1)
									
Age	65 to 69	1.4 (1.2–1.6)	1.4 (1.1–1.6)	1.3 (1.1–1.6)	1.5 (1.2–1.9)	2.0 (1.2–3.2)	2.1 (1.3–3.4)	2.2 (1.3–3.6)	2.3 (1.4–3.9)
	70 to 74	1.1 (0.9–1.3)	1.02 (0.9–1.2)	0.99 (0.8–1.2)	0.99 (0.8–1.2)	1.6 (0.9–2.6)	1.5 (0.9–2.6)	1.7 (0.96–3.1)	1.8 (0.95–3.5)
									
VISITS TO A GENERAL PRACTITIONER									
Total		1.3 (1.1–1.4)	1.2 (1.1–1.4)	1.2 (1.1–1.3)	1.3 (1.1–1.5)	1.4 (1.02–1.8)	1.4 (1.1–1.9)	1.4 (1.03–1.9)	1.3 (0.9–1.8)
									
Age	65 to 69	1.4 (1.2–1.7)	1.4 (1.2–1.6)	1.4 (1.2–1.6)	1.5 (1.3–1.8)	1.5 (0.99–2.1)	1.5 (1.04–2.2)	1.5 (1.03–2.3)	1.4 (0.9–2.2)
	70 to 74	1.1 (0.97–1.3)	1.04 (0.9–1.2)	1.03 (0.9–1.2)	1.04 (0.9–1.3)	1.2 (0.8–1.9)	1.2 (0.8–1.9)	1.2 (0.7–1.9)	1.1 (0.7–1.9)
									
VISITS TO A SPECIALIST									
Total		0.8 (0.8–0.9)	0.8 (0.7–0.9)	0.7 (0.7–0.8)	0.8 (0.7–0.9)	1.3 (1.00–1.6)	1.3 (1.02–1.6)	1.3 (0.98–1.7)	1.3 (0.95–1.7)
Age	65 to 69	0.9 (0.8–1.04)	0.9 (0.8–1.04)	0.8 (0.7–0.9)	0.8 (0.7–0.9)	1.3 (0.96–1.8)	1.4 (1.00–1.9)	1.3 (0.9–1.9)	1.4 (0.97–2.1)
	70 to 74	0.8 (0.7–0.9)	0.7 (0.7–0.9)	0.7 (0.6–0.8)	0.8 (0.6–0.9)	1.2 (0.8–1.7)	1.1 (0.8–1.7)	1.2 (0.8–1.7)	1.1 (0.7–1.6)
									
HOSPITAL CARE ^2^									
Total		0.7 (0.6–0.7)	0.6 (0.6–0.7)	0.6 (0.5–0.7)	0.6 (0.5–0.7)	1.00 (0.8–1.3)	1.00 (0.8–1.3)	0.9 (0.7–1.3)	0.8 (0.6–1.2)
									
Age	65 to 69	0.7 (0.6–0.8)	0.7 (0.6–0.8)	0.6 (0.5–0.7)	0.6 (0.5–0.7)	0.97 (0.7–1.4)	1.01 (0.7–1.5)	0.9 (0.6–1.4)	0.9 (0.5–1.4)
	70 to 74	0.6 (0.6–0.7)	0.6 (0.5–0.7)	0.6 (0.5–0.7)	0.6 (0.5–0.7)	1.00 (0.7–1.5)	0.98 (0.6–1.5)	0.96 (0.6–1.5)	0.8 (0.5–1.4)
									
VISITS TO A PHYSIOTHERAPY									
Total		1.7 (1.5–2.0)	1.6 (1.4–1.9)	1.4 (1.3–1.7)	1.5 (1.3–1.7)	1.1 (0.8–1.5)	1.1 (0.8–1.5)	1.04 (0.8–1.4)	1.1 (0.8–1.5)
									
Age	65 to 69	1.5 (1.3–1.8)	1.5 (1.2–1.8)	1.3 (1.1–1.6)	1.4 (1.1–1.7)	1.7 (1.1–2.6)	1.7 (1.1–2.6)	1.8 (1.1–2.8)	1.9 (1.1 -3.1)
	70 to 74	1.9 (1.6–2.4)	1.8 (1.5–2.2)	1.6 (1.3–2.0)	1.7 (1.3–2.1)	0.7 (0.4–1.1)	0.7 (0.4–1.1)	0.6 (0.4–0.98)	0.6 (0.4–1.1)

^1 ^Nord-Tröndelag in Norway, Helsinki Metropolitan area and North-Karelia in Finland

^2 ^In Norway: during the past 5 years, In Finland: during the past 12 months.

Model 1: adjusted for age and (in Finland) region

Model 2: adjusted for age, self-rated health and (in Finland) region

Model 3: adjusted for age, self-rated health, self-reported chronic diseases and (in Finland) region

Model 4: adjusted for age, self-rated health and self-reported chronic diseases, level of education, marital status, and (in Finland) region.
